# Antidepressant and Anxiolytic Effects and Subacute Toxicity of the Aerial Parts of *Psychotria ankasensis* J.B.Hall (Rubiaceae) in Murine Models

**DOI:** 10.1155/2021/5543320

**Published:** 2021-05-18

**Authors:** Francis Ackah Armah, Isaac Tabiri Henneh, Isaac Kingsley Amponsah, Robert Peter Biney, Fynn Malcolm, John Alake, Wisdom Ahlidja, Mustapha Abubakar Ahmed, Christian Kweku Adokoh, Genevieve Etornam Adukpo, Desmond Omane Acheampong, Peter K. Gathumbi

**Affiliations:** ^1^Department of Biomedical Sciences, School of Allied Health Sciences, University of Cape Coast, Cape Coast, Ghana; ^2^School of Pharmacy and Pharmaceutical Sciences, University of Cape Coast, Cape Coast, Ghana; ^3^Department of Pharmacognosy, Faculty of Pharmacy and Pharmaceutical Sciences, KNUST, Kumasi, Ghana; ^4^Department of Chemistry, School of Physical Sciences, University of Cape Coast, Cape Coast, Ghana; ^5^Department of Forensic Sciences, School of Biological Sciences, University of Cape Coast, Cape Coast, Ghana; ^6^Department of Veterinary Pathology, Microbiology and Parasitology, Faculty of Veterinary Medicine, University of Nairobi, Nairobi, Kenya

## Abstract

**Background:**

The present study aimed at validating the traditional use and toxicity profile of a methanolic extract of the aerial parts of *Psychotria ankasensis* in alleviating depression and anxiety disorders.

**Method:**

The antidepressant effect of methanolic extract of *Psychotria ankasensis* (PAE 30, 100, and 300 mg/kg, *p.o*.) was assessed in mice using the forced swim test (FST) and the tail suspension test (TST). The plant's anxiolytic potential was also evaluated in mice using the elevated plus-maze (EPM) and the open field tests (OFT). The subacute toxicity was assessed via oral administration of PAE at doses of 100, 300, and 1000 mg/kg in rats for 28 days.

**Results:**

PAE 100 and 300 mg/kg showed antidepressant-like properties by significantly (at least *p* < 0.05) decreasing the frequency and duration of immobility in FST and TST. PAE (100 and 300 mg/kg) also showed a significant (at least *p* < 0.05) anxiolytic effect in both EPM and OFT. In the EPM test, *E*_max_ for PAE and diazepam were 92.52 ± 40.11% and 85.95 ± 45.92%, respectively, whereas *E*_max_ was approximately 100% for both test drugs in the OFT. Subacute administration of PAE did not reveal any toxic effects with respect to organ weight index, haematological, serum biochemical, and histopathological parameters.

**Conclusions:**

Methanolic extract of *P. ankasensis* exhibited antidepressant-like and anxiolytic-like effects devoid of significant toxicity at the doses tested in murine models.

## 1. Background

Depression is one of the most common mental disorders that affects one out of every sixteen persons worldwide [1]. It is characterized by symptoms such as anhedonia, low self-esteem, and suicidality. It is a leading cause of a number of diseases or disability burden all over the world [2]. Antidepressants in clinical use, such as the selective serotonin reuptake inhibitors (SSRIs) and tricyclic antidepressants (TCAs), are refractory in about 50% of patients, have delayed onset of action, and are accompanied by numerous side effects [3]. Besides, a high comorbidity exists between depression and anxiety, two mental diseases that reduce patients' quality of life [4].

Anxiety disorder is a prevalent psychiatric condition that occurs in one-eighth of the world's population making it a prominent research area in psychopharmacology [5]. Anxiety disorders possess a very high disease, social, and economic burden [6]. The use of medications such as benzodiazepines, SSRIs, beta-blockers, and monoamine oxidase inhibitors in treating anxiety disorders is limited by numerous side effects [7, 8]. For instance, the sudden withdrawal of benzodiazepines causes withdrawal syndrome characterized by olfactory and tactile disturbance, disorientation, and paresthesia [9]. These shortcomings create the need for alternative medications for anxiety and depression.

Over 2000 plant species within the Psychotria genus have been reported to affect the central nervous system (CNS) [10]. For instance, monoterpene indole alkaloids isolated from *Psychotria suterella* and *Psychotria laciniata* were found to significantly inhibit monoamine oxidases (MAO-A and MAO-B) obtained from rat brain mitochondria [11, 12]. The inhibition of the monoamine oxidase enzymes constitutes an important mechanism of action for antidepressants in the monoamine oxidase inhibitor class such as phenelzine [13]. A scientific study of *Psychotria* genus indicates that several species within this genus can mitigate neurodegenerative disorders in addition to their analgesic, antimutagenic, antioxidant, antiviral, antipyretic, and anti-inflammatory activities [10, 14]. In Ghana, the indigens within the communities surrounding the Ankasa Forest where the plant was first identified traditionally use *Psychotria ankasensis* aerial parts to treat infectious diseases [15]. Research on its medicinal properties is not widespread, and it is traditionally used in the treatment of anxiety and depression in Ghana. The present study considered the effects of *Psychotria ankasensis* methanolic extract on anxiety and depression as well as elucidating its toxicity profile in rodents.

## 2. Results

### 2.1. PAE Produced an Antidepressant-Like Effect in the Forced Swim Test

**Figure 1:**
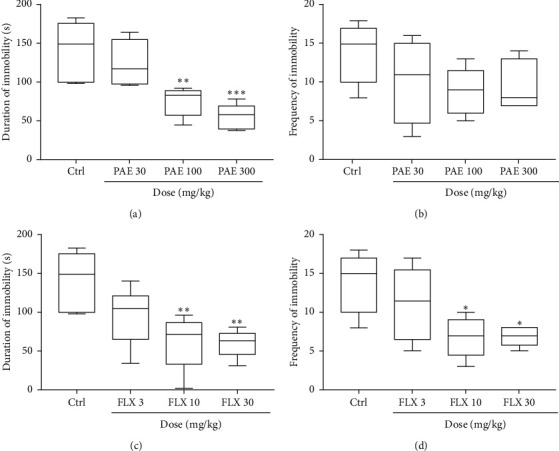
Effects of PAE (30, 100, and 300 mg/kg) treatment on the (a) duration and (b) frequency of immobility versus the effect of fluoxetine (FLX: 3, 10, and 30 mg/kg) on (c) duration and (d) frequency of immobility of mice in the forced swim test. Animals in the control group (Ctrl) received vehicle water. Data are presented as group means ± SEM (*n* = 7). ^*∗∗∗*^*p* < 0.001, ^*∗∗*^*p* < 0.01, and ^*∗*^*p* < 0.05 (one-way ANOVA followed by Tukey's post hoc comparison test).

**Figure 2:**
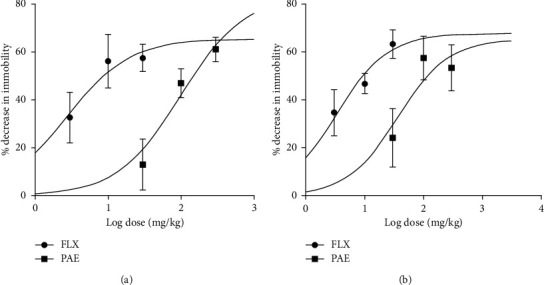
Dose-response curves of PAE and fluoxetine administration: (a) forced swim test and (b) tail suspension test in mice.

PAE 100 and 300 mg/kg significantly (*p* < 0.01 and *p* < 0.001, respestively) decreased the duration of immobility in the FST, but the frequency of immobility was not significantly altered (Figures 1(a) and 1(b)). Fluoxetine also produced a significant (*p* < 0.01) decrease in immobility duration at 10 and 30 mg/kg (Figures 2(a) and 2(b)). Dose-response curves plotted produced ED_50_ values of 99.21 ± 1.83 and 2.71 ± 2.17 mg/kg for PAE and FLX, respectively (Figure 2(a)). The maximum efficacy (*E*_max_) values were 83.69 ± 19.16 and 65.66 ± 12.56% for PAE and fluoxetine, respectively (see Figure 2(a)).

### 2.2. PAE Produced an Antidepressant Effect in the Tail Suspension Test

**Figure 3:**
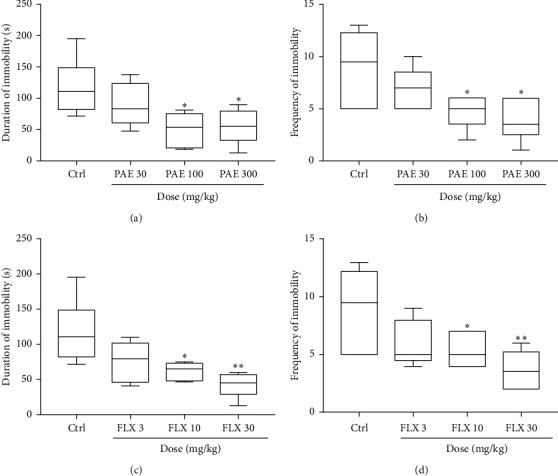
Effects of PAE (30, 100, and 300 mg/kg) treatment on the (a) duration and (b) frequency of immobility of mice versus the effect of fluoxetine (FLX: 3, 10, and 30 mg/kg) on (c) duration and (d) frequency of immobility in the tail suspension test. Animals in the control group (Ctrl) received vehicle. Data are presented as group means ± SEM (*n* = 7). ^*∗∗∗*^*p* < 0.001, ^*∗∗*^*p* < 0.01, and ^*∗*^*p* < 0.05 (one-way ANOVA followed by Tukey's post hoc comparison test).

The oral administration of PAE 100 and 300 mg/kg resulted in a significant (*p* < 0.05) decrease in immobility duration and frequency in the tail suspension test (Figure 3). Fluoxetine, 10 mg/kg (*p* < 0.05) and 30 mg/kg (*p* < 0.01) increased the duration and frequency of immobility compared to the control group. ED_50_ values obtained from the dose-response curves showed that the extract (ED_50_ = 36.28 ± 2.30) was less potent than fluoxetine (ED_50_ = 3.28 ± 1.75) (Figure 2(b)). On the other hand, the maximal efficacy (*E*_max)_ values of the extract and fluoxetine (Figure 2(b)) were 65.49 ± 9.72% and 67.81 ± 15.32%, respectively.

### 2.3. PAE Exerted Anxiolytic-Like Effect in the Elevated Plus-Maze Test

**Figure 4:**
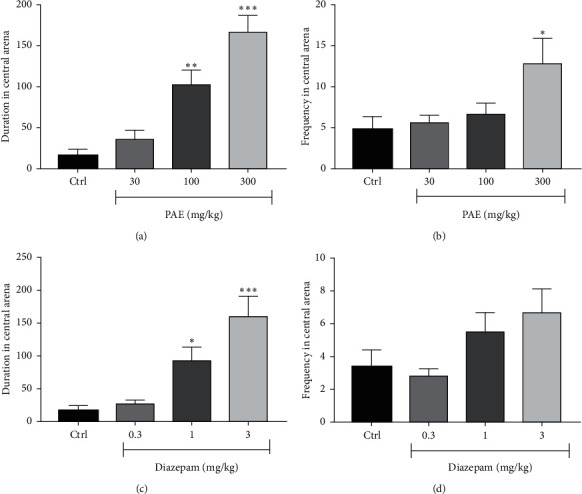
Effects of PAE (30, 100, and 300 mg/kg) treatment on the (a) duration and (b) frequency of entry of mice into the open arms of the elevated plus-maze test versus the effect of diazepam (0.3, 1, and 3 mg/kg) on (c) duration and (d) frequency of entry of mice into the open arms of the elevated plus-maze test. Animals in the control group (Ctrl) received only vehicle. Data are presented as group means ± SEM (*n* = 7). ^*∗∗∗*^*p* < 0.001, ^*∗∗*^*p* < 0.01, and ^*∗*^*p* < 0.05 (one-way ANOVA followed by Tukey's post hoc comparison test).

**Figure 5:**
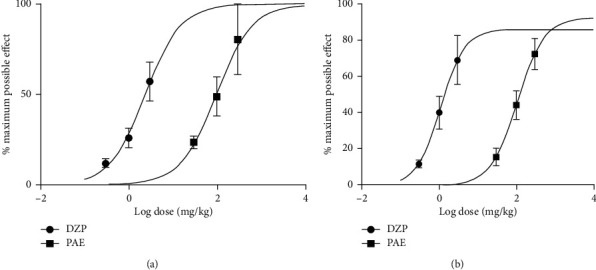
Dose-response curves of PAE and diazepam (DZP) administration in the (a) open field tests in mice and (b) elevated plus-maze.

Results illustrated in Figure 4 show that there was a significant change in open arm entry between treatment groups in the elevated plus-maze test. The administration of PAE at 100 and 300 mg/kg significantly (*p* < 0.01 and *p* < 0.01, respectively) increased the duration mice spent in the open arms of the EPM compared to the control group (see Figure 4(a)). A similar observation was made with diazepam 1 mg/kg (*p* < 0.05) and 3 mg/kg (*p* < 0.001) as presented in Figure 4(c). The frequency of entry into the open arms was also significantly (*p* < 0.05) increased at 300 mg/kg of PAE (Figure 44(b)), whereas diazepam could not increase it significantly (Figure 4(d)). ED_50s_ for PAE and diazepam were 107.30 ± 4.45 and 2.71 ± 1.10 mg/kg, respectively. On the other hand, *E*_max_ for PAE and diazepam were 92.52 ± 40.11% and 85.95 ± 45.92%, respectively (Figure 5(b)).

### 2.4. PAE Produced an Anxiolytic-Like Effect in the Open Field Test

**Figure 6:**
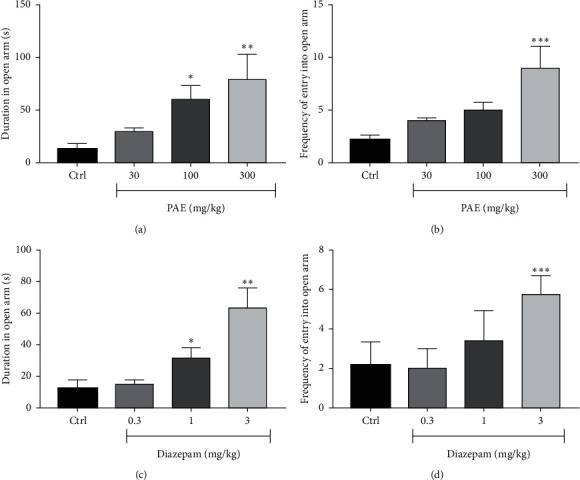
Effects of PAE (30, 100, and 300 mg/kg) treatment on the (a) duration and (b) frequency of entry of mice into the central arena of the open field versus the effect of and diazepam (0.3, 1, and 3 mg/kg) on the (c) duration and (d) frequency of entry of mice into the central arena of the open field. Animals in the control group (Ctrl) received only vehicle. Data are presented as group means ± SEM (*n* = 7). ^*∗∗∗*^*p* < 0.001, ^*∗∗*^*p* < 0.01, and ^*∗*^*p* < 0.05 (one-way ANOVA followed by Tukey's post hoc comparison test.

In the open field test, both PAE 100 mg/kg (*p* < 0.05) and 300 mg/kg (*p* < 0.01) and diazepam 1 mg/kg (*p* < 0.05) and 3 mg/kg (*p* < 0.01) significantly increased the duration mice spent in the open arms of the OFT compared to the untreated animals. The frequency of entry into the open arena was also significantly (*p* < 0.001) increased by the highest doses of both PAE and diazepam (Figure 6). ED_50_ values obtained for PAE and diazepam in the test were 96.77 ± 4.43 and 2.37 ± 1.30, respectively. *E*_max_ values were, however, approximately 100% for both test drugs (Figure 5(a)).

### 2.5. Subacute Toxicity Evaluation of PAE

All the animals survived the 28-day experimental period. No notable physical changes were observed in any of the animals. Although body weight gain was observed across all treatment groups, there were no statistically significant differences between the treatment groups and the control group (Table 1). The average raw weights and weight indices of vital organs such as heart, liver, spleen, brain, kidneys, and lungs were not significantly different from the control group (Tables 2 and 3). Moreover, the haematological and biochemical analysis conducted on the animals after treatment with PAE (100, 300, and 1000 mg/kg, *p.o*.) revealed no significant (*p* > 0.05) differences when compared with the control group (Tables 4 and 5).

#### 2.5.1. Histopathology

**Figure 7:**
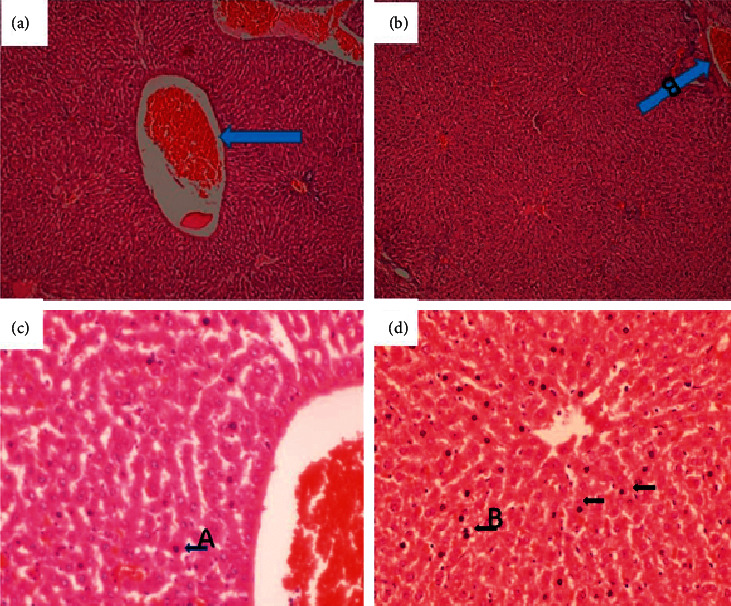
Liver of a rat that was orally administered 1,000 mg/kg methanolic extract of *Psychotria ankasensis* (H&E x100 (a), x400 (c)). The structure of the liver is normal, but blood vessels are moderately distended with blood (congested, see (a) broader arrow). Most hepatocytes are normal in structure and are characterized by abundant pink cytoplasm and a large rounded vesicular nucleus (low chromatin density). A few scattered hepatocytes show single-cell necrosis characterized by a condensed, dark-stained nucleus and reduced cytoplasm that stains more eosinophilic (pinker) than in normal hepatocytes (see (c) arrow). Liver of a rat that was administered vehicle as control (H&E x100 (b), x400 (d) shows that the liver is structurally well preserved and predominantly normal; blood vessels are moderately distended (image (b), arrow); a few scattered hepatocytes have a condensed dark-stained nucleus and a reduced amount of cytoplasm that stains more eosinophilic (pinker) are randomly scattered in the organ (see image (d), arrows). These are scattered dead hepatocytes that represent mild, randomly distributed, single-cell hepatocellular necrosis.

**Figure 8:**
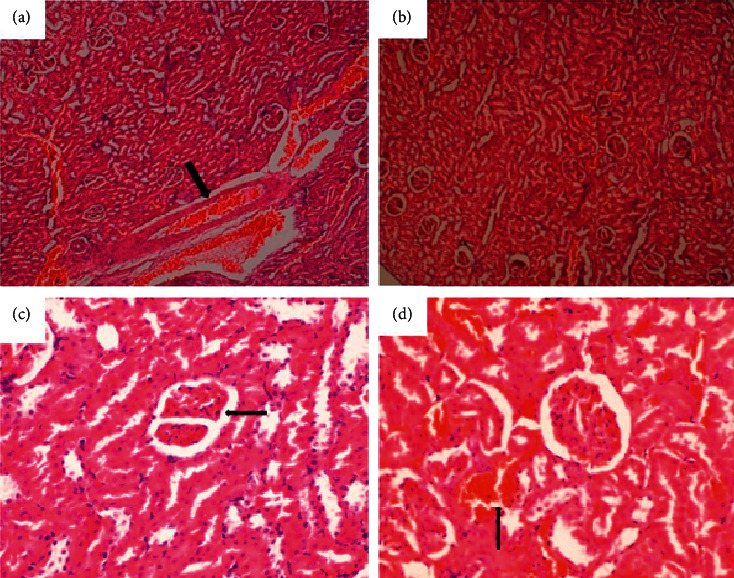
Kidney of a rat that was orally administered 1,000 mg/kg methanolic extract of *Psychotria ankasensis* (H&E x100 (a), x400 (c)). Renal tubules, glomeruli ((c) arrow), and interstitial tissues are well preserved, but blood vessels are moderately distended with blood (congested, (a) arrow) versus kidney of a control rat that was experimentally administered vehicleas control (H&E, x100 (b)), x400 (d). Tubule, glomeruli, and interstitial tissues have a normal structure. There is mild distension of blood vessels (congestion, see image (d) arrow).

**Figure 9:**
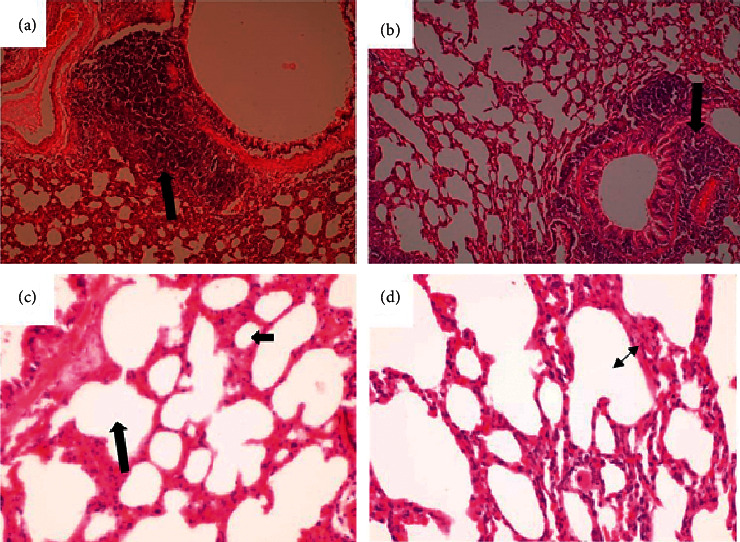
Lung of a rat that was orally administered 1,000 mg/kg methanolic extract of *Psychotria ankasensis (*H&E x100 (a) x400 (c)) daily for 28 days. Alveoli are empty, but some are expanded (emphysema), while others are reduced in volume (atelectasis); see image (c), and the interalveolar wall is thicker in some parts of the lung than in others (image (c)). Comparatively, the lung of a rat that was administered with vehicle as control (H&E x100 (b)), x400 (d), shows the structure of the lung is well preserved, some alveoli have larger volume (emphysema, image (d), double arrow), while others are collapsed as is described for other groups. This is considered to be a nonlesion as long as the animals had no clinical respiratory signs. Peribranchial lymphoid tissue is prominent (see arrow in images (a) and (b)).

**Figure 10:**
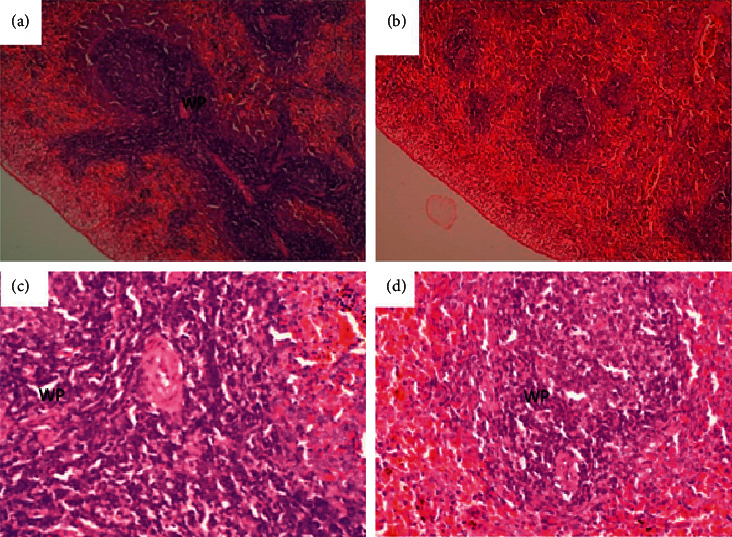
Spleen of a rat (that was orally administered 1,000 mg/kg methanolic extract of *Psychotria ankasensis* for 28 days (H&E, x100 (a), x400 (c)). White pulp has high lymphoid cell density in Malpighian follicles, and the red pulp has normal structure. The spleen of a control rat administered distilled (H&E x100 (b), x400 (d)) shows prominent white pulp (WP) with high lymphoid cell density, and the red pulp has a normal structure.

(1) There were no significant gross and histopathology lesions that could be attributed to the effects of the plant extract in the liver. Most hepatocytes had a normal structure with a large vesicular nucleus and abundant pink cytoplasm. However, a few hepatocytes that were sparsely spread in the tissue contained a dark condensed nucleus and a more eosinophilic (pinker) and less abundant cytoplasm. This was consistent with single hepatocyte necrosis (death), in the midst of many normal ones, across the entire liver (Figure 7). This represents mild randomly distributed single-cell hepatocellular necrosis. The biliary system, vasculature, and supportive tissues and cells of the defense system in the liver were in normal status. There were moderate distensions of few blood vessels, consistent with mild congestion (Figure 7). These observations occurred in the test and control groups and could therefore not be linked with the effects of the extract.

(2) Renal histology revealed no abnormalities in test and control. The structure of the tubules, glomerulus, and interstitial tissues was normal in the entire kidney (Figure 8). A few scattered blood vessels were moderately distended with blood (congested). Parenchyma had normal architecture, and all tissues were in apparently normal morphology.

(3) The lungs showed no abnormalities. Across the tissue, alveoli (arrow) were empty in some parts of the tissue, but some had a slightly larger volume (emphysema). In contrast, others were smaller (atelectasis), and the interalveolar wall was thicker and had more cell density. These are considered as nonlesions since they also occurred in control animals. Bronchial-associated lymphoid tissue (BALT (P)) was prominent. Morphological features of the tissues were the same as in tissues from control animals (Figure 9).

(4) The spleen, brain, and heart showed no abnormalities. The morphology of the tissues in the spleen was similar to that of control animals. In all cases, the red and white pulp was normal in structure, the latter being densely populated with lymphoid cells in Malpighian follicles (Figure 10).

**Figure 11:**
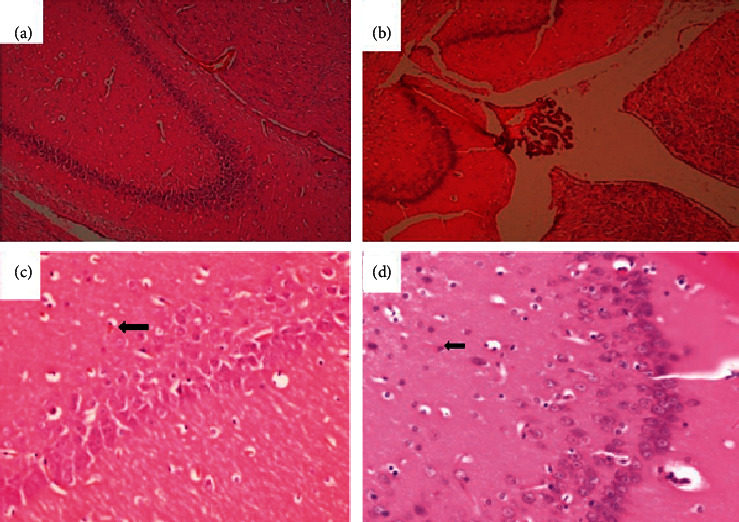
Brain of a rat that was orally administered 1,000 mg/kg methanolic extract of *Psychotria ankasensis* (H&E x 100 (a), x 400 (c)). Brain structure is normal. However, while most neurons are normal in structure, a few scattered ones show condensed eosinophilic cytoplasm and dark, shrunken nucleus. This change is consistent with acute ischemic neuronal injury, (C) arrow). The brain of a rat administered with vehicle as control (H&E, x100 (b), x400 (d)) shows that majority of neurons and glial cells are well preserved, and the organ is predominantly normal. One or a few neurons show acute ischemic neuronal injury characterized by the condensed nucleus and reduced cytoplasm that stains more eosinophilic (pinker, image D, arrow).

**Figure 12:**
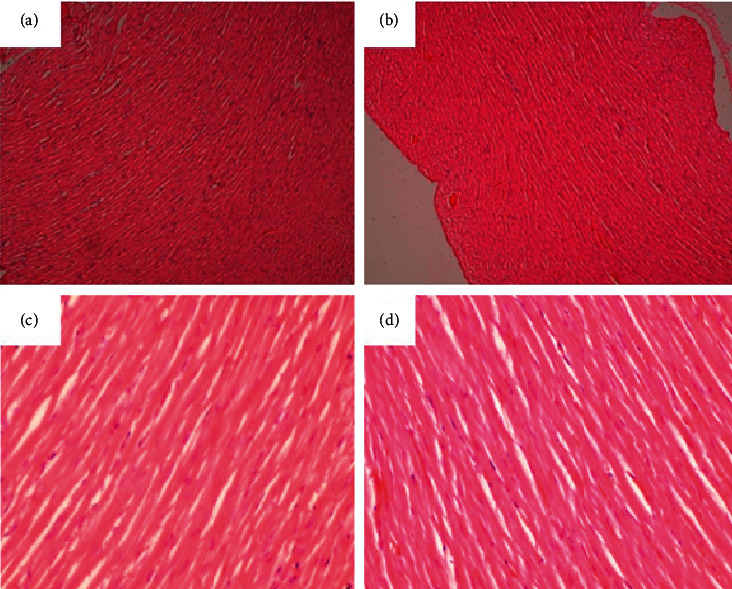
Heart of a rat that was orally administered 1,000 mg/kg methanolic extract *Psychotria ankasensis* daily for 28 days (H&E x100 (a), x400 (c)). The myocardium is normal, as are the intermuscular tissues. Heart of a control rat that was experimentally administered with the vehicle as a control (H&E x100 (b), x400 (d)). Myocardium and intercellular tissues have a normal structure.

A section through the midbrain showed a typical Ammon's horn of the hippocampus (broad arrow) with a normal superstructure (Figure 11). A few neurons individually had condensed nucleus and cytoplasm that stains eosinophilic (c and d arrow), a change that was consistent with an acute ischemic injury of affected neurons. Coincidentally, it also occurred in both treatment and control groups. Therefore, as in the liver, it could not be associated with the extract but may be an external factor, probably acute anoxia during euthanasia. Additionally, the heart showed no abnormalities as the structure of the myocardium and intermuscular tissues appeared normal in the treatment and control groups (Figure 12).

### 2.6. Phytochemical Analysis

**Figure 13:**
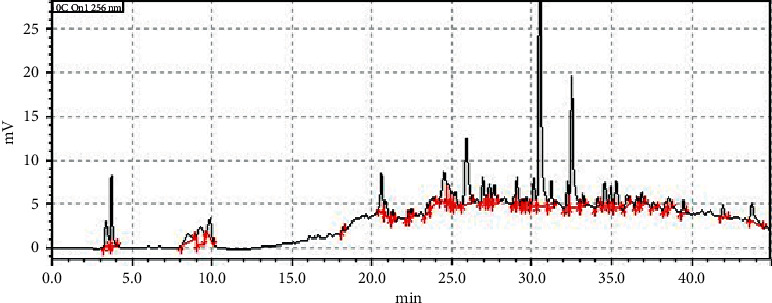
HPLC fingerprint of PAE measured at a wavelength of 256.

*Psychotria ankasensis* was found to contain alkaloids, triterpenoids, steroids, tannins, anthraquinone glycosides, saponins, and reducing sugars (Table 6). In an attempt to standardise the extract to afford repeatability or further study, an HPLC chromatogram was developed (Figure 13). The HPLC fingerprint showed 45 peaks as presented (Figure 13 and Table 7). Two significant peaks (with retention times of 30.495 and 32.485 minutes) were selected as marker peaks for the identification of *Psychotria ankasensis.* A detailed result showing the retention times, areas under the curves, heights, and areas (%) has been supplied in Table 7.

## 3. Discussion

*Psychotria ankasensis* showed marked antidepressant-like activity. It significantly (at least *p* < 0.05) reversed the increased duration of immobility induced by the stressful situation of forced swimming and tail suspension test (Figures 1–3). The extract exhibited similar efficacy as the 1^st^ line antidepressant drug, fluoxetine, in both the tail suspension and forced swim test. This highlights the potential of the extract towards developing and validating indigenous plant remedies for depression and anxiety.

The forced swim and tail suspension tests were employed in these tests as they have been commonly used in depression research due to their high predictive potential [16]. Both tests utilise the principle of behavioural despair, which is similar to human depression as the animals become immobile following numerous unsuccessful attempts to escape from the inescapable condition. The tests' validity is based on the fact that almost all the antidepressants in clinical use significantly decrease the duration and frequency of immobility in both tests [16]. The effect observed within the study suggests it could be a source of novel phytochemicals which could be isolated and characterized to provide valuable leads for optimization and further development in the antidepressant drug discovery pipeline.

Anxiety disorder is reported to be the most predominant comorbid condition of depression [17]. Antidepressants, for example, the selective serotonin reuptake inhibitors, are also known to exhibit a significant anxiolytic effect. Therefore, the extract's anxiolytic actions were assessed using the elevated plus-maze and the open field tests.

The elevated plus-maze is used to test mice's aversion towards falling from a high unprotected arm of the maze. This approach-avoidance test exploits the innate exploratory drive of rodents against their natural fear of unprotected environments. A test compound's ability to tilt this balance towards favouring the exploratory drive with minimal anxiety is predictive of potential anxiolytic-like effects [18]. Thus, administration of benzodiazepine and other anxiolytics inreaseses the exploration of the unprotected arms of the maze by mice treated with anxiolytics compared to naivemice [19]. As observed in the study, the administration of PAE and diazepam an hour before the test resulted in a significant increase in duration (*p* < 0.01) and frequency (*p* < 0.05) of entry into the unprotected well-illuminated open arms of the test (Figure 4). This gives an indication that PAE is a potential anxiolytic agent.

In the open field test, the introduction of the mice to a relatively larger, well-illuminated, and novel arena compared to their breeding or natural environment is known to induce anxiety-like behaviours such as increased thigmotaxis, defecation as well as increased time spent in the closed dark corners. The ability of test compounds to increase the time spent in the centre of the apparatus and the frequency of central arena entry but not the corners or periphery has been recognised as an anxiolytic effect [7, 8]. This was confirmed by diazepam (Figure 6), which is an established anxiolytic drug in clinical use. PAE also significantly (*p* < 0.05) increased the duration and frequency of entry into the centre arena, just as was observed with diazepam (Figure 6). This gives further confirmation of an anxiolytic-like effect and confirms the results seen in the EPM. PAE actually exhibited a comparable efficacy as diazepam, the current first-line drug for treating many types of anxiety (Figure 5). This gives credence to the plant's traditional usage and reveals its potential for further exploration for novel phytocompounds with anxiolytic activity.

With the antidepressant-like and anxiolytic-like efficacy of *P. ankasensis* established, there was the need to assess its toxicity since safety is the highest cause of attrition in the drug discovery and development pipeline. There were no statistically significant differences between the organ weights of the liver, kidney, lungs, spleen, and heart of the treated groups compared to the control group (Table 2) in the subacute activity. Similarly, the average weight indices of all the organs assessed did not change significantly (Table 3). It is known that toxic substances' consumption can substantially alter the weight indicies of these vital organs [20]. Therefore, the insignificant changes give an early indication that the plant extract may not be toxic to these essential organs.

PAE's apparent safety was further confirmed by evaluating serum biochemical, gross anatomical, and histological markers of organ damage. Haematological and biochemical parameters evaluation constitute an essential indicator in evaluating xenobiotic toxicity in animals [21–23]. Results obtained from the haemogram showed the extract exerted no deleterious effect on whole blood cell count parameters such as PCV, MCH, MCV, MCHC, and haemoglobin in the treated groups compared to the control group. Also, RBC, Hb, PCV, MCV, MCH, and MCHC were all not significantly affected by the treatment. The absence of any significant changes on RBC, MCV, MCHC, PCV, MCH, and HCT may be because there were no changes in the red blood cells' erythropoiesis and morphology, as well as the integration of haemoglobin into red blood cells [24]. Also, there were no significant differences in platelet, lymphocytes, monocytes, and neutrophils count in the treated group compared to the control group. These insignificant changes in haematological parameters are an indication of the safety of the test substances, as has been suggested in other studies [25].

Biochemical parameters such as serum electrolytes and other secretory enzymes and proteins constitute additional vital biomarkers for determining the functional capacity of the various organs of the body [23]. For instance, the liver is known to play a crucial role in drug metabolism. As such, alterations in these biochemical parameters can significantly affect the normal function of the liver [24]. Alternationin lipid and plasma enzymes such as AST (aspartate aminotransferase) and ALT (alanine aminotransferase) constitute an important index for identifying injured hepatocytes. At the same time, the elevation of ALP (alkaline phosphatase) and GGT (gamma-glutamyl transferase) in serum indicates liver injuries that cause bile stasis [26]. However, the present study results did not show any significant alterations in the tested liver biochemical enzyme markers that could be attributed to the subacute administration of *P. ankasensis* extract. Again, bilirubin is an important endogenous organic anion obtained from haemoglobin breakdown. It is a product of haem's metabolic degradation from senescent red blood cells and is one of the commonly used marker in liver function test [27]. The present study indicates no statistical difference in bilirubin levels between the control group and the treatment groups. It also worth-mentioning that most serum proteins (other than immunoglobulins), such as albumin, fibrinogen, *α*, and *β* globulins, and other coagulation factors are synthesized from the parenchymal cells of the liver [28, 29]. Quantitatively, albumin is among the vital plasma proteins synthesized by the liver and is used to evaluate liver function [24]. The result showed no statistical difference between the treatment group and the control groups. These findings were supported by the histopathological examination of the liver (Figure 7), which revealed that most of the hepatocytes showed a regular architecture with a large vesicular nucleus and abundant pink cytoplasm. Although mild single-cell hepatocellular necrosis was seen in the liver tissues of few rats in both treatment and control groups, most hepatocytes were normal. As such, the liver function would not be affected by such mild single-cell necrosis of hepatocytes. Generally, liver function decreases if more than 75% of hepatocytes are dead. Since liver tissue from control animals also showed the same response as in other groups of animals, the observed mild necrosis of hepatocytes could not have been caused by the extract but an external factor which is likely to be the euthanizing agent.

Again, renal function parameters are used to evaluate the normal functioning of different sections of the nephrons [30]. Similarly, the serum concentrations of uric acid, electrolytes, creatinine, and urea provide an understanding of the toxic effects produced by test substances on the tubular and glomerular functions of the kidney [24]. Kidney enzymes and biochemical markers such as creatinine, urea, uric acid, and electrolytes (Table 4) observed in the experimental group were not significantly different from the control group (*p* > 0.05). Histopathological examination of the kidney (tubules, glomerulus, and interstitial tissues) revealed no abnormality as PAE treated groups were similar to the control group (Figure 8).

The present study (Figure 9) also showed that *P. ankasensis* could not induce any pathological alterations in the tissues of the lungs of the experimental group as no abnormalities were seen. Although the treatment groups' alveoli appeared empty in some parts of the tissue, with some having considerably larger lumen while others were smaller. Also, the interalveolar wall seemed thicker. All these could be considered nonlesions since they also occurred in the control group. Also, Bronchial-Associated Lymphoid Tissue (BALT) was prominent in all groups. Morphological features of the tissues were the same as in tissues from the control group.

Histological examination of haematoxylin and eosin-stained tissues from the spleen of animals treated with *P. ankasensis* indicated no tissue morphology abnormalities. Since no significant difference was seen in the treated group compared to the control group, it suggests that *P*. *ankasensis* has no damaging effect on the spleen (Figure 10).

Based on the brain's histopathological result (Figure 11), the study did not reveal any damage to the midbrain. Also, the structures of the brain are normal. However, few neurons have a condensed nucleus and cytoplasm that show eosinophilic changes consistent with an acute ischemic injury of affected neurons. Since it was observed in sections of the treated group and the control group, it cannot be associated with the *P. ankasensis* extract. Still, it may be an external factor such as anoxia associated with euthanasia.

Changes in concentrations of significant lipids such as cholesterol, low-density lipoproteins (LDL), high-density lipoprotein (HDL), and triglycerides provide a useful indicator of cardiac function [30] as it is associated with the predisposition of the heart to atherosclerosis and its attended cardiac complications. High levels of lipids except HDL are associated with increased risk of atherosclerosis with elevated LDL levels, and triglycerides are associated with coronary artery disease [21]. In this study, there was no significant (*p* > 0.05) difference in the lipid profile of the treatment groups compared to the control group (Table 4). Also, no abnormal pathological alterations were detected during the histopathological examination of the heart. The morphology of tissues of the heart in the treated groups was not different from the control group (Figure 12).

Phytochemicals such as alkaloids, triterpenoids, steroids, tannins, anthraquinone glycosides, saponins, and reducing sugars were present in the plant. The HPLC chromatogram of the extract developed (Figure 13) will serve as a template for tracking the extract for further research or quality control of any potential phytomedicine from the plant. The presence of these phytochemicals has been shown to account for the efficacy and toxicity of medicinal plants [31, 32]. Specifically, *β*-carboline and monoterpene indoles alkaloids isolated from several *Psychotria* species have been shown to produce inhibitory properties against monoamine oxidase proteins, which are enzymatic targets depression and anxiety [33]. As a result, the pharmacological activity observed in this study can be attributed to the presence of the above phytochemicals. Our further research will focus on isolating the compounds responsible for the observed effect. Also, the mechanisms underpinning the observed anxiolytic-like and antidepressant-like effect will be explored in chronic animal models.

## 4. Conclusion

*P. ankasensis* extract exhibited antidepressant and anxiolytic effects in murine models. There were no significant toxic effects associated with the doses of the extract used in this study.

## 5. Materials and Methods

### 5.1. Plant Material Collection

Aerial parts of *Psychotria ankasensis* were collected from the Ankasa forest (5°17′00.0″N, 2°39′00.0″W) in Jomoro District, Western Region of Ghana in February 2019. The plant was identified and authenticated by a botanist at the Herbarium of School of Biological Sciences, University of Cape Coast, and a voucher specimen preserved (identification number FAA/DR/003).

### 5.2. Preparation of the Extract

The aerial parts of *Psychotria ankasensis* were air-dried for three weeks and then pulverized using a hammer mill to obtain a fine powder. An amount of 1200 g of the powdered sample was weighed and cold macerated with 4000 mL of absolute methanol for 72 h. It was filtered and the resulting filtrate concentrated using a rotary evaporator at a reduced temperature of 40˚C. The extract was further dried in a desiccator containing activated silica until it was thoroughly dried to to obtain a dark gummy extract (PAE, 84.1 g). It was subsequently stored in a refrigerator, and the required doses were reconstituted immediately before drug administration.

### 5.3. Drugs and Chemicals

Methanol was obtained from the British Drug House (Poole, UK), whereas diazepam, fluoxetine, tween 80, and other reagents used were of analytical grade bought from Sigma-Aldrich Inc., St. Louis, MO, USA. Emulsions of the extract, fluoxetine and diazepam were prepared using tween 80 *q.s*. as a surfactant. Control group animals received vehicle which comprised 1% tween 80 in distilled water. The selection of doses and times of administration were based on the findings from preliminary studies on the extract.

### 5.4. Experimental Animals

Male Sprague-Dawley rats (8–10 weeks old, 190-240 g), as well as Imprint Control Region (ICR) mice (7 weeks old, 20-25 g), were used in this experiment. These animals were purchased from the Noguchi Memorial Institute for Medical Research (NMIMR), Ghana, and kept at the Department of Biomedical Sciences' animal house, University of Cape Coast (UCC). They were acclimatised for two weeks before the experiments under standard laboratory conditions of room temperature (25 ± 1°C) and relative humidity (40 ± 10%) with a 12/12 h light/dark cycle. The animals were housed in stainless steel cages with softwood shaving as bedding, cleaned, and maintained daily and fed with standard commercial pelleted rodent feed (Floor Mills of Ghana Limited, Tema, Ghana) and water *ad libitum* during acclimatisation and experimental periods. It was ensured that doses of extract, positive control drugs, and vehicle did not exceed 1 mL/100 g body weight (for mice) or 2 mL/100 g body weight (for rats). The National Institute of Health Guidelines for the Care and Use of Laboratory Animals were adhered to throughout the study. All procedures employed in this study were approved by the University of Cape Coast Ethical Review Board (clearance number UCCIRB/CHAS/2018/14).

### 5.5. Evaluation of the Antidepressant Effect of PAE

#### 5.5.1. Forced Swim Test (FST)

A method initially described was adopted for the study [34]. Forty-nine (49) mice were randomly grouped into seven (*n* = 7). One hour after PAE 30, 100, or 300 mg/kg, fluoxetine 3, 10, or 30 mg/kg or vehicle10 mL/kg administration using oral gavage, mice were carefully placed individually into transparent plastic cylinders (height = 30 cm; diameter = 15 cm), filled with water (25 ± 1°C) up to a depth of 20 cm. Each mouse was allowed to swim for 6 min period and captured with a camcorder hanged above the cylinder. The mice were removed from the water after each test session, dried with a towel, and placed near a heat source until they were completely dried. The duration and frequency of immobility during the last 4 min of the test were quantified using JWatcher software (Version 1.0^TM^).

#### 5.5.2. Tail Suspension Test (TST)

The test was performed following a protocol described [16]. Briefly, mice were randomly assigned to seven groups (*n* = 7) and given PAE 30, 100, or 300 mg/kg, fluoxetine 3, 10, or 30 mg/kg, or vehicle10 mL/kg orally via a gavage. One hour after administration of the various treatments, the tails of the mice were suspended on a horizontal bar mounted 50 cm from a bench. After the suspension on the bar (i.e., climbing and swinging), the movement of the mice was recorded for 6 min with a camcorder. The duration and frequency of immobility of the mice for the entire 6 min session were tracked and quantified using the JWatcher Version 1.0^®^ software.

### 5.6. Evaluation of the Anxiolytic-Like Effect of PAE

#### 5.6.1. Elevated Plus-Maze (EPM) Test

A procedure previously described [35] was used with some modifications. Briefly, the apparatus was made up of a typical central square platform (5 × 5 cm) with an extension of two fenced arms (15 × 5 × 30 cm^3^) facing each other and another extension of two unfenced arms perpendicular to the fenced arms (15 × 5 cm^2^). To prevent the mice from falling off the unfenced arm of the maze, a 0.5 cm rim of Plexiglas enclosed the borders of the open arms. The whole apparatus was elevated 50 cm above the ground. The mice were divided into seven groups of seven mice each and pretreated with either PAE 30, 100, or 300 mg/kg, *p.o.* or the reference drug, diazepam 0.3, 1, and 3 mg/kg, *p.o* orvehicle, 10 mL/kg; *p.o.* The mice were placed individually in the centre of the maze facing the open arm at the start of the experiment. Their behavior was videotaped for 5 mins using a digital camera placed 75 cm above the maze and tracked for frequency duration spent in the open or closed arms using JWatcher.

#### 5.6.2. Open Field Test (OFT)

A previously described method was followed [18]. Sixty minutes after oral administration of the various treatment to their respective groups, as indicated in the EPM test above, mice were placed individually in the centre of the open field. The apparatus was made of a wooden box (60 × 60 × 60 cm^3^) divided into 16 squares (15 × 15 cm^2^) and well illuminated. The four inner squares in the centre were deemed centre arena. The remaining 12 squares on the periphery, along the walls, and corners were considered a peripheral arena. Each mouse was recorded for 5 min with a camera. The frequency and time spent in each arena were tracked and quantified using the JWatcher.

### 5.7. Subacute Toxicity Evaluation of PAE

An earlier described method [36] was followed. Twenty Sprague-Dawley rats were randomly grouped into four groups (*A*, *B*, *C*, and *D*) of five rats each. Group A animals were orally administered with 10 mL/kg of vehicle once daily for 28 days, whereas groups *B*, *C*, and *D* received PAE at 100 mg/kg, 300 mg/kg, and 1000 mg/kg, respectively, for 28 days. The rats were observed twice daily for morbidity and mortality throughout the 28 days. All the animals had free access to food and water. They were weighed on days 0, 14, and 28 using a digital weighing balance (Sartorius Lab Instruments GmbH & Co. Goettingen, Germany). On the 29^th^ day, Euthanasia was achieved by inhalation method as follows. Cotton soaked with diethyl ether was placed in a clean glass chamber and covered to achieve saturation within the chamber. The animals were then placed in the chamber one animal at a time and covered until total loss of consciousness was achieved. Loss of consciousness was rapid and painless. On average, the onset of sedation was 5 seconds. Blood samples were collected from the left ventricle of the heart. An amount of 1 ml of the blood was collected into EDTA tubes for the haematological analyses. Another 4 mL of blood was collected into gel separator tubes for biochemistry analyses.

#### 5.7.1. Haematological Analysis

Blood samples were analysed for erythrocyte count (RBC), haemoglobin (Hb), haematocrit (HCT), mean corpuscular haemoglobin (MCH), mean corpuscular haemoglobin concentration (MCHC), mean corpuscular volume (MCV), platelets count (PLC), leukocyte count (WBC), and their differentials using the autohaematology analyser (URIT-5250Vet, China).

#### 5.7.2. Biochemical Analysis

Samples collected for biochemistry analyses were centrifuged at 10 mins at 3000 rpm to obtain the serum. The serum obtained was assayed for the lipid profile, total proteins, albumin, globulins, alanine aminotransferase (ALT), alkaline phosphatase (ALP), aspartate aminotransferase (AST), creatinine, urea, and blood urea nitrogen using an auto biochemistry analyser (URIT-8021AVet, China).

#### 5.7.3. Necropsy and Histology of Vital Organs

On the 29^th^ day of the experiment, surviving animals were weighed and then euthanised for autopsy. Animals were put under heavy sedation by diethyl ether inhalation as described above in 5.7. A complete systematic autopsy was conducted, and gross lesions were recorded. Vital organs, namely, kidneys, liver, spleen, lungs, heart, and brain, were weighed using a digital weighing balance (Sartorius Lab Instruments GmbH & Co. Goettingen, Germany). Samples for histopathology were taken from organs with gross lesion and the following vital organs, kidneys, liver, spleen, lungs, heart, and brain, and fixed in 10% phosphate-buffered formalin for 24 hrs. Fixed tissues were processed for routine histopathology, embedded in paraffin, sectioned at 4-5 *µ*m, and stained in haematoxylin and eosin [37]. Sections were examined using an Olympus microscope mounted with a live view digital SLR camera (E-330), and photomicrographs were taken.

### 5.8. Phytochemical Screening

The qualitative phytochemical test was performed on the *Psychotria ankasensis* powder according to methods described by Harborne [38].

### 5.9. HPLC Fingerprint of the Extract

A Shimadzu LC-20AD with SPD- 20AV UV-VIS Detector was used for the test. The mobile phase comprised 0.10 M acetic acid: acetonitrile using gradient elution. The percentages and times of varying the acetonitrile have been supplied in Table 8. A solution of 10 mg/mL solution of PAE was constituted using methanol. A volume of 10 *µ*L of the extract (10 mg/mL) was injected into the columns and allowed to run for 45 min at a flow rate of 0.5 mL/min, under a pump pressure of 1700 Psi and a temperature of 40°C. The chromatograms obtained were detected at a wavelength of 256 nm and the retention times, areas under curve, concentrations, heights, and areas (%) of the chromatograms were then determined.

### 5.10. Statistical Analysis

Data has been presented as mean ± standard error of the mean (SEM). Graphpad® Prism Version 7.0 (GraphPad Software, San Diego, CA, USA) for Windows was used to perform all statistical analysis with *p* < 0.05 considered statistically significant for all tests. Differences between treatment groups were determined using one-way analysis of variance followed by Tukey's post hoc test. Dose-response curves were plotted using an iterative curve fitting method which follows a nonlinear regression four-parameter logistic equation as follows:

(1) $$\begin{array}{c} y=d+\frac{a-d}{1+{\left(x/c\right)}^{b}}, \end{array}$$

where y is the dependent variable and x is the independent variable. The four parameters used were *a*  is  the minimum value that can be obtained, *b* is Hill's slope of the curve, *c* is the point of inflection, and *d* is the maximum value that can be obtained. With the aid of the above equations, ED_50s_ were estimated using GraphPad® Software, San Diego, CA, USA.

### 5.11. Limitation of the Study

Authors do believe that this study is not without limitations and that the data presented therein should be interpreted with the challenges in view. Firstly, acute rodent behavioural models were employed in the study which are highly predictive models for depression and anxiety. The models do not induce significant structural modifications in the brain within the short time; hence, our decision not to perform histopathological/immunohistochemical assessment of brain areas implicated in depression. Our further research focuses on the effect of the extract in chronic models of depression and anxiety. Secondly, ether anaesthesia was employed as euthanasia procedure in the study. Although this method has some ethical implication due to its irritatory effect, we are also aware that all other euthanasia methods have their limitations. We ensured that any effect reported in the study was not attributable to the euthanasia method by subjecting all animals in the control and treatment groups to the same euthanasia method.

## Figures and Tables

**Table 1:** Average body weight (g) of the Sprague-Dawley rats.

Days	Control	PAE 100 mg/kg	PAE 300 mg/kg	PAE 1000 mg/kg
0	233.53 ± 2.40	223.22 ± 3.01	213.03 ± 2.12	224.1 ± 2.03
14	237.05 ± 4.10	221.71 ± 1.21	221.71 ± 2.90	275.52 ± 4.70
28	244.33 ± 5.33	228.52 ± 5.40	229.00 ± 3.80	231.13 ± 6.30

Results are presented as Mean ± SEM (*n* = 5). One-way analysis of variance of all the datasets revealed no statistical significance (*p* > 0.05).

**Table 2:** Average organ weight (g) of the Sprague-Dawley rats aged 12–14 weeks.

Parameter (g)	Control	PAE 100 mg/kg	PAE 300 (mg/kg)	PAE 1000 mg/kg
Heart	0.82 ± 0.05	0.62 ± 0.06	0.82 ± 0.03	0.74 ± 0.07
Liver	6.57 ± 0.28	5.18 ± 0.34	6.86 ± 0.36	6.06 ± 0.27
Spleen	0.75 ± 0.03	0.73 ± 0.11	0.91 ± 0.09	0.81 ± 0.06
L. kidney	0.64 ± 0.04	0.53 ± 0.02	0.63 ± 0.05	0.59 ± 0.05
R. kidney	0.64 ± 0.15	0.52 ± 0.04	0.69 ± 0.05	0.60 ± 0.04
Lungs	1.94 ± 0.17	1.38 ± 0.03	1.77 ± 0.08	1.42 ± 0.14
Brain	1.76 ± 0.11	1.81 ± 0.21	1.76 ± 0.04	1.82 ± 0.32

Results are presented as mean ± SEM (*n* = 5). One-way analysis of variance of all the datasets revealed no statistical significance (*p* > 0.05).

**Table 3:** Average organ weight index of the Sprague-Dawley rats aged 12–14 weeks.

Parameter	Control	PAE 100 mg/kg	PAE 300 (mg/kg)	PAE 1000 mg/kg
Heart	0.0034 ± 0.0002	0.0027 ± 0.0002	0.0036 ± 0.0002	0.0032 ± 0.0002
Liver	0.0269 ± 0.0011	0.0227 ± 0.0015	0.0300 ± 0.0016	0.0262 ± 0.0012
Spleen	0.0031 ± 0.0001	0.0032 ± 0.0005	0.0040 ± 0.0004	0.0035 ± 0.0003
Left kidney	0.0026 ± 0.0002	0.0023 ± 0.0001	0.0027 ± 0.0002	0.0026 ± 0.0002
Right kidney	0.0027 ± 0.0001	0.0023 ± 0.0002	0.0030 ± 0.0002	0.0026 ± 0.0002
Lungs	0.0079 ± 0.0007	0.0060 ± 0.0001	0.0077 ± 0.0004	0.0062 ± 0.0006
Brain	0.0072 ± 0.0002	0.0074 ± 0.0001	0.0072 ± 0.0003	0.0074 ± 0.0001

Organ weight index = organ weight/carcass weight. Results are presented as mean ± SEM (*n* = 5). One-way analysis of variance of all the datasets revealed no statistical significance (*p* > 0.05).

**Table 4:** Results from the haematological parameters analyses of the control and treated groups.

Parameter	Control	PAE 100 mg/kg	PAE 300 (mg/kg)	PAE 1000 mg/kg
WBC (10^9^/L)	14.92 ± 0.29	14.75 ± 1.65	15.75 ± 1.11	15.60 ± 0.29
LYM (%)	46.85 ± 5.84	66.79 ± 4.43	57.43 ± 4.79	56.69 ± 4.13
MON (%)	8.72 ± 1.58	10.96 ± 0.10	17.80 ± 0.27	19.82 ± 2.60
NEU (%)	37.45 ± 5.05	25.21 ± 8.39	22.90 ± 4.92	22.67 ± 2.70
EOS (%)	6.44 ± 1.02	2.50 ± 1.64	1.73 ± 0.49	3.15 ± 0.4542
BASO (%)	0.36 ± 0.09	0.05 ± 0.01	0.20 ± 0.05	0.28 ± 0.04
LYM# (10^9^/L)	2.05 ± 0.46	6.21 ± 3.40	2.98 ± 0.72	2.81 ± 0.43
MON# (10^9^/L)	0.55 ± 0.22	0.99 ± 0.50	1.12 ± 0.13	0.82 ± 0.12
NEU# (10^9^/L)	1.04 ± 0.10	1.65 ± 0.62	1.53 ± 0.29	1.08 ± 0.09
EOS# (10^9^/L)	0.18 ± 0.01	0.15 ± 0.04	0.10 ± 0.03	0.15 ± 0.02
BASO# (10^9^/L)	0.01 ± 0.00	0.01 ± 0.00	0.01 ± 0.00	0.01 ± 0.01
RBC (10^12^/L)	7.89 ± 0.08	8.07 ± 0.21	8.83 ± 0.60	8.61 ± 0.23
HGB (g/dL)	14.92 ± 0.29	14.75 ± 1.65	15.75 ± 1.11	15.60 ± 0.29
HCT (%)	37.76 ± 0.78	36.80 ± 3.30	40.68 ± 3.70	39.75 ± 1.00
MCV (fL)	47.94 ± 0.82	45.65 ± 2.95	45.88 ± 1.26	46.23 ± 0.30
MCH (pg)	18.86 ± 0.36	18.20 ± 1.60	17.78 ± 0.45	18.1 ± 0.37
MCHC (g/dL)	39.6 ± 0.24	40.00 ± 1.00	39.00 ± 1.08	39.25 ± 0.75
RDW (%)	12.04 ± 0.25	13.05 ± 0.85	14.05 ± 0.32	13.20 ± 1.64
RDW_SD (fL)	44.62 ± 1.38	42.50 ± 1.10	46.25 ± 1.96	42.35 ± 2.61
RDW_CV (%)	12.04 ± 0.26	13.05 ± 0.85	14.05 ± 0.32	13.2 ± 1.64
PLT (10^9^/L)	1041.00 ± 60.97	1281.00 ± 574.50	1341.00 ± 142.50	961.00 ± 86.27
MPV (fL)	4.70 ± 0.1924	5.30 ± 0.20	4.65 ± 0.09	4.33 ± 0.16
PDW (fL)	6.88 ± 0.24	8.05 ± 2.35	7.50 ± 0.39	6.25 ± 0.30
PCT (%)	0.48 ± 0.03	0.69 ± 0.33	0.62 ± 0.07	0.41 ± 0.04

Results are presented as mean ± SEM, *p* < 0.05 (n = 5). One-way analysis of variance of all the datasets revealed no statistical significance (*p* > 0.05). WBC: white blood cell count, LYM: lymphocytes, MON: monocytes, NEU: neutrophils, EOS: eosinophils, BASO: basophils, RBC: red blood cell count, HGB: haemoglobin, HCT: haematocrit, MCH: mean corpuscular haemoglobin, MCHC: mean corpuscular haemoglobin concentration, MCV: mean corpuscular volume, PLT: platelets count, RDW: red blood cell distribution width, RDW_CV: red blood cell distribution width coefficient of variation, MPV: mean platelet volume, PDW: platelet distribution width, and PCT: plateletcrit.

**Table 5:** Results from serum biochemical parameters analyses of the various groups.

Parameter	Control	PAE 100 mg/kg	PAE 300 (mg/kg)	PAE 1000 mg/kg
TBil (*µ*mol/L)	8.17 ± 0.24	9.06 ± 0.70	8.69 ± 0.78	7.86 ± 0.44
Dbil (*µ*mol/L)	3.62 ± 0.42	3.57 ± 0.24	3.78 ± 0.20	3.38 ± 0.19
TP (g/L)	70.48 ± 2.49	69.8 ± 1.29	73.40 ± 3.33	79.66 ± 4.65
ALB (g/L)	28.90 ± 0.74	29.1 ± 1.52	29.10 ± 2.01	30.70 ± 0.51
GLB (g/L)	39.50 ± 1.30	40.70 ± 2.00	44.33 ± 1.99	44.38 ± 1.54
A/G	0.65 ± 0.08	0.72 ± 0.06	0.65 ± 0.05	0.62 ± 0.08
ALT (U/L)	54.51 ± 4.60	59.49 ± 7.30	67.92 ± 6.32	71.08 ± 5.15
ALP (U/L)	79.56 ± 1.30	77.55 ± 4.75	75.53 ± 4.45	78.24 ± 3.23
AST (U/L)	95.02 ± 9.12	116.30 ± 16.08	113.40 ± 18.50	99.68 ± 8.79
GGT (U/L)	6.2 ± 0.58	7.67 ± 1.33	6.50 ± 1.56	7.60 ± 0.40
UREA (mmol/L)	6.96 ± 0.50	5.77 ± 0.66	7.28 ± 1.31	6.76 ± 0.89
CREAT(*µ*mol/L)	145.00 ± 2.76	119.30 ± 10.93	130.10 ± 17.83	134.70 ± 4.39
UA (*µ*mol/L)	33.66 ± 3.72	30.48 ± 2.40	27.33 ± 2.38	28.30 ± 3.60
GLU (*µ*mol/L)	7.586 ± 0.88	11.04 ± 1.77	7.79 ± 1.11	11.20 ± 1.38
Ca (*µ*mol/L)	2.54 ± 0.02	2.64 ± 0.08	2.69 ± 0.06	2.58 ± 0.01
LDL (*µ*mol/L)	0.64 ± 0.08	0.72 ± 0.03	0.72 ± 0.07	0.64 ± 0.05
TG (*µ*mol/L)	1.01 ± 0.12	0.69 ± 0.09	1.03 ± 0.19	0.84 ± 0.09
CHOL (*µ*mol/L)	1.79 ± 1.00	1.58 ± 0.07	2.11 ± 0.14	1.82 ± 0.11
HDL C (*µ*mol/L)	0.63 ± 0.05	0.56 ± 0.03	0.88 ± 0.03	0.75 ± 0.08

Results are presented as Mean ± SEM (*n* = 5): one-way analysis of variance of all the datasets revealed no statistical significance (*p* > 0.05). TBill: total bilirubin, DBill: direct bilirubin, TP: total proteins, ALB: albumin, GLB: globulins, ALT: alanine aminotransferase, ALP: alkaline phosphatase, AST: aspartate aminotransferase, CREAT: creatinine, UA: uric acid, GLU: glucose, and TG: triglycerides, CHOL: cholesterol, LDL: low-density lipoprotein, HDL C: high-density lipoprotein C, Ca: calcium, GGT: gamma GT, and A/G: albumin-to-globulins ratio.

**Table 6:** Phytochemical analysis of *Psychotria ankasensis*.

Constituents	Present/absent
Alkaloids	+
Terpenoids	+
Anthraquinone aglycones	+
Fatty acids	+
Reducing sugars	+
Tannins	+
Saponins	+
Steroids	+
Flavonoids	−

The symbols (+) indicates present and (−) indicates absent.

**Table 7:** Table showing the retention times, areas under the curves, heights, concentrations, and areas (%) of the various peaks in the HPLC fingerprint of PAE.

Peak#	Ret. time	Area	Height	Mark	Conc.	Area (%)
1	3.373	31961	2929		2.099	2.099
2	3.689	54117	8203	*V*	3.553	3.553
3	8.524	24647	916	*M*	1.618	1.618
4	9.271	47466	2033	*M*	3.117	3.117
5	9.871	30385	2260		1.995	1.995
6	18.193	3677	503		0.241	0.241
7	20.602	44920	4509		2.95	2.95
8	20.874	19943	1690	*V*	1.309	1.309
9	21.316	11228	1259		0.737	0.737
10	22.281	7354	987		0.483	0.483
11	22.448	8963	1085	*V*	0.589	0.589
12	23.45	11403	1042		0.749	0.749
13	23.854	18140	937	*V*	1.191	1.191
14	24.51	56966	3490		3.741	3.741
15	24.817	29272	2127	*V*	1.922	1.922
16	25.05	12836	1356	*V*	0.843	0.843
17	25.22	18122	1653	*V*	1.19	1.19
18	25.923	111862	7551		7.345	7.345
19	26.928	28772	2932		1.889	1.889
20	27.208	7013	925	*V*	0.461	0.461
21	27.357	20418	2159	*V*	1.341	1.341
22	27.657	22195	2026	*V*	1.457	1.457
23	28.908	9098	1093		0.597	0.597
24	29.079	28267	3185	*V*	1.856	1.856
25	29.494	6033	813		0.396	0.396
26	29.673	8832	1033	*V*	0.58	0.58
27	30.128	31287	3123	*V*	2.054	2.054
28	30.495	340727	33958	*V*	22.373	22.373
29	31.218	27536	2954	*V*	1.808	1.808
30	32.235	26739	3160		1.756	1.756
31	32.485	179902	15071	*V*	11.813	11.813
32	33.192	12452	1417	*V*	0.818	0.818
33	34.103	9859	1037		0.647	0.647
34	34.538	26553	2713		1.744	1.744
35	34.954	22226	2299	*V*	1.459	1.459
36	35.29	33589	2997		2.206	2.206
37	35.953	16057	1226		1.054	1.054
38	36.586	11935	1182		0.784	0.784
39	36.887	13520	1411	*V*	0.888	0.888
40	37.612	8098	664		0.532	0.532
41	38.325	9676	871		0.635	0.635
42	38.547	18507	1311	*V*	1.215	1.215
43	39.465	13556	1454		0.89	0.89
44	41.948	12418	1284		0.815	0.815
45	43.766	34407	1952		2.259	2.259
Total		1522938	138778		100	100

**Table 8:** Table showing the procedure for the gradient elution.

Time	% composition of acetonitrile
0.00	5
2.10	5
28.00	95
35.40	95
35.50	5
45.00	Stop

## Data Availability

All data generated or analysed during this study are included within this manuscript.

## Abbreviations

EPM: Elevated plus-maze
FLX: Fluoxetine
FST: Forced swim test
H&E: Hematoxylin and eosin
NMIMR: Noguchi Memorial Institute for Medical Research
OFT: Open field test
PAE: *Psychotria ankasensis* extract
TST: Tail suspension test.
